# Magnesium Oxide Nanoparticles: Effective Agricultural Antibacterial Agent Against *Ralstonia solanacearum*

**DOI:** 10.3389/fmicb.2018.00790

**Published:** 2018-04-25

**Authors:** Lin Cai, Juanni Chen, Zhongwei Liu, Hancheng Wang, Huikuan Yang, Wei Ding

**Affiliations:** ^1^Laboratory of Natural Product Pesticide, College of Plant Protection, Southwest University, Chongqing, China; ^2^Guizhou Key Laboratory of Agro-Bioengineering, Guizhou University, Guiyang, China; ^3^Guizhou Academy of Tobacco Science, Guiyang, China

**Keywords:** MgONPs, tobacco, *Ralstonia solanacearum*, antibacterial mechanism, control efficacy

## Abstract

Magnesium (Mg) is an essential mineral element for plants and is nontoxic to organisms. In this study, we took advantage of nanotechnologies to systematically investigate the antibacterial mechanisms of magnesium oxide nanoparticles (MgONPs) against the phytopathogen *Ralstonia solanacearum* (*R. solanacearum*) *in vitro* and *in vivo* for the first time. *R. solanacearum* has contributed to catastrophic bacterial wilt, which has resulted in the world-wide reduction of tobacco production. The results demonstrated that MgONPs possessed statistically significant concentration-dependent antibacterial activity, and the minimum inhibitory concentration (MIC) and minimum bactericidal concentration (MBC) were measured as 200 and 250 μg/mL, respectively. Additional studies, aimed at understanding the toxicity mechanism of MgONPs, indicated that physical injury occurred to the cell membranes, along with decreased motility and biofilm formation ability of *R. solanacearum*, due to the direct attachment of MgONPs to the surfaces of the bacterial cells, which was observed by scanning electron microscopy (SEM) and transmission electron microscopy (TEM). Reactive oxygen species (ROS) accumulation could also be an important reason for the antibacterial action, inducing DNA damage. The toxicity assessment assay under greenhouse conditions demonstrated that the MgONPs had exerted a large effect on tobacco bacterial wilt, reducing the bacterial wilt index. Altogether, the results suggest that the development of MgONPs as alternative antibacterial agents will become a new research subject.

## Introduction

Nanotechnology, as a burgeoning interdisciplinary area of research in a variety of fields, has the potential to enable breakthrough applications in agriculture regarding plant protection and nutrition, which involve pesticide delivery, nanosensors, pesticide degradation, micronutrients for efficient use, etc. (Ghormade et al., 2011). To the best of our knowledge, due to their unusual superior physicochemical properties, high surface-to-volume ratio and unique nanosize structure characteristics, several inorganic and organic metal oxide nanomaterials, and several hybrid nanomaterials, such as TiO_2_, ZnO, CuO (Kalhapure et al., 2015), graphene oxide (Chen et al., 2014), and Fe_3_O_4_-Ag core shell magnetic nanoparticles (Hemeg, 2017), are being increasingly applied as alternative antibacterial agents in biomedical applications. Recent investigations have demonstrated that they exhibit strong antimicrobial activity toward the pathogenic bacteria *Streptococcus mutans* (Liu et al., 2014) and *Xanthomonas perforans* (Paret et al., 2013), fungi *Fusarium graminearum* (*F. graminearum*) (Liu et al., 2017) and a few viruses (Mishra et al., 2011). With their high pertinence to antibacterial applications, the use of nanoparticles for the prevention and control of plant diseases is a promising and valuable topic because of their increased effectiveness, durability and, particularly, their high specific surface area, which can stimulate interactions with living cells (Kang et al., 2008). Further, the previous literature also noted that the inorganic metal nanoparticles (such as ZnO, Ag, TiO_2_, Cu) are being increasingly applied as antimicrobial, owing to the accumulation of reactive oxygen species (ROS), which could damage cellular components, such as proteins, lipids, even nucleic acids (Hemeg, 2017).

Among the many different inorganic metal oxides, magnesium oxide nanoparticles (MgONPs) are an antibacterial agent with the advantages of being nontoxic and relatively easy to obtain. MgONPs have been recognized as safe materials by the United States Food and Drug Administration (21CFR184.1431). Recent advances have led to conspicuous developments with enormous potential in materials and medicines (Krishnamoorthy et al., 2012b). For example, MgONPs can relieve heartburn, initiate post-activation of bone repair scaffolds and act as hyperthermia agents in cancer therapy (Martinez-Boubeta et al., 2010). More recently, Bindhu et al. (2016) specifically found that MgONPs have extremely pronounced antibacterial activities against *Staphylococcus aureus* (*S. aureus*) in culture media. Previous studies demonstrated that the peptide linkages in *Pseudomonas aeruginosa* and *Escherichia coli* cell membrane were destroyed by the generation of superoxide ions on the surface of MgONPs (Huang et al., 2005). MgONPs can distort and damage the cell membranes of *E. coli*, resulting in the leakage of their intracellular content and eventually death (Jin and He, 2011). However, little is known about the antimicrobial properties of MgONPs toward plant pathogenic bacteria. Moreover, the detailed mechanism of MgONPs as bactericidal agents and their ability to expectantly control plant disease are not yet clear. Given the prospect of employing nanomaterials in agriculture, it is important to mention here that the increasing concern regarding the toxicity of MgONPs to environmental systems cannot be ignored. In all cases, the effectiveness of these nanoparticles is directly related to their antimicrobial activity and the ability to affect as few plant cells as possible.

Bacterial wilt, which first occurred in temperate areas, such as Europe and North America, is a devastating plant disease worldwide that is caused by the aggressive phytopathogen *Ralstonia solanacearum*. *R. solanacearum* is a soil-borne and nonsporing bacterium that can infect several hundred host plant species around the world, including potatoes, tomatoes, eggplants, groundnuts, olives, bananas, and ginger (Schell, 2000). The pathogens sense specific stimuli, move toward the plants’ roots by swimming and attaching to the roots, then, cluster on the xylem vessels and block the vascular system by excessively secreting cell-wall-degrading enzymes and extracellular polysaccharides (EPS) within the plant tissue, eventually inducing host death (Danhorn and Fuqua, 2007; Bogino et al., 2013). There are several treatments for controlling bacterial wilt, including breeding resistance varieties, chemical control, and biocontrol (Yuliar et al., 2015). Breeding of resistant varieties is the most effective measure for disease management, but this method is labor intensive; thus, most high-quality varieties of tobacco in production are not resistant to the bacterial wilt of tobacco. Unfortunately, a few massive threats and environmental risks caused by the inappropriate and unreasonable application of control treatments have emerged. Hence, the development of effective technological innovations to meet these large global challenges is urgently needed.

Fortunately, investigations have indicated that MgONPs induce systemic resistance against *R. solanacearum* by activating the salicylic acid (SA-), jasmonic acid (JA-), and ethylene (ET-) signaling pathways in tomato plants (Imada et al., 2016). These facts highlight the possibility of using MgONPs as an efficient alternative to chemical pesticides in crop protection. To realize the objectives of applying MgONPs, we recently demonstrated the antibacterial activity of MgONPs against phytopathogenic *R. solanacearum*, the main culprit of the catastrophic bacterial wilt disease and, for the first time, illuminated its interaction mechanism with MgONPs. Herein, the bactericidal experiments were conducted using several technologies (including transmission electron microscopy (TEM), scanning electron microscopy (SEM), and scanning confocal laser microscopy) to investigate the main toxicity process between MgONPs and *R. solanacearum.* The synergistic effects between the membrane disruption of the cell, the oxidative stress, and the inhibition of biofilms, as well as motility activity, are proposed, which are related to the physiological, ultrastructural, and virulence-related characteristics of *R. solanacearum*. The ability to further control the efficiency of MgONPs for preventing tobacco bacterial wilt by root-zone irrigation and the side effects of MgONPs on tobacco in a greenhouse were investigated.

## Materials and Methods

### Characterization of Nanoparticles

The MgONPs and bulk MgO were purchased from Sigma-Aldrich (Sigma) Chemical Co. The crystalline structure and morphology of the MgONPs were observed by TEM (JEM-2100, JEOL, Japan) and SEM (S-570, Hitachi, Japan), respectively. The MgONPs were suspended in an excess amount of ethanol (100%), mounted on TEM copper grids and scanned at 200 kV. The particle size distribution and zeta potential of the MgONP dispersions were evaluated using a Malvern Zetasizer Nano Series (Malvern, United Kingdom).

### Bacterial Strains and Growth Conditions

*Ralstonia solanacearum* (biovar 3, phylotype I) was isolated from naturally infected tobacco in Pengshui (Chongqing, China), which was identified as a highly pathogenic strain. The separated bacteria were streaked on a triphenyl tetrazolium chloride (TTC) medium at 30°C, and a single colony was selected for culturing in B medium per 1 L medium containing Difco Bacto-Peptone (10 g), Difco yeast extract (1 g), and Difco casamino acids (1 g) at 30°C overnight at 200 g. Then, these cultures were harvested during the logarithmic phase and washed at least three times with sterilized water by centrifugation until the medium residue was removed. The cell cakes were resuspended in deionized water, and the suspension concentration was adjusted to the optical density (OD = 1.0) at 600 nm.

### Determination of the Minimum Inhibitory Concentration (MIC) and Minimum Bactericidal Concentration (MBC)

To investigate the antibacterial activity of the MgONPs and bulk MgO, these materials were diluted in a series of concentrations ranging from 15.625 to 1000 μg/mL (1000, 500, 250, 125, 62.5, 31.25, and 15.625 μg/mL), and a typical microdilution method was performed to determine the MIC and MBC of these chemicals against *R. solanacearum* (Consoli et al., 2018). The MIC and MBC were determined to indicate the bacteriostatic and bactericidal activity.

Specifically, a 100 μL suspension (1000 μg/mL MgONPs or bulk MgO in B medium) was added to 96-well polystyrene microtiter plates and then diluted with B medium in a geometric progression to obtain the final test concentrations mentioned above. A mixture of sterilized water and B medium served as the control. Then, 1 μL of cultured bacteria (OD_600_ = 1.0) and 1 μL of TTC were inoculated in each well filled with the MgONPs or bulk MgO suspension. Afterward, the plates were kept at 30°C without shaking. The concentrations of the cells were monitored by an ELISA microplate reader. Measurements of the optical density at 600 nm (OD_600_) were made for different incubation times (24, 48, and 72 h). And the same method was also suitable for determining the MIC and MBC of thiodiazole copper.

### Cell Viability Measurement

Referring to a previous study (Tiwari et al., 2017), viable bacteria were determined by a colony counting method, and fresh *R. solanacearum* cells were diluted to 10^5^ CFU (the number of colony-forming units)/mL. For the toxicity assessment, 100 μL was sampled and directly inoculated onto the casamino acid peptone glucose (CPG) agar medium plates filled with different concentrations of the antibacterials MgONPs (50, 100, 150, 200, and 250 μg/mL), bulk MgO (50, 100, 150, 200, and 250 μg/mL), and thiodiazole copper (50, 100, 150, 200, and 250 μg/mL). After the cells were cultured 3 days in an incubator, the CFU was counted on the agar plates. Each set of experiments was repeated four times to ensure data reproducibility. The cell viability was calculated according to the following formula:

$$\mathrm{Cell}\;\mathrm{viability}\;(\%)=\frac{A_{1}}{A}\times 100\%,$$

where A denotes the number of colony-forming units on the control plates and A_1_ denotes the number of colony-forming units after using different concentrations of the drug treatment.

### Growth Curve Assay

The growth curve was defined as the logarithm of the relative population size as a function of time, which usually reflected the vitality of the bacteria. Here, the curve was used to assess the antibacterial activity of the nanomaterials (Karahan et al., 2016). The growth curve of *R. solanacearum* was measured by the optical density (OD_600_) during 26 h of cultivation. Specifically, the pure bacterial suspension was inoculated in 30 mL of rich B medium in a culture flask, and then, 200 μL of the nanoparticles or bulk MgO was added to the miscible liquids as mentioned above to obtain the following concentrations: 50, 100, 150, 200, and 250 μg/mL. The same volume of deionized water was used in the B medium for the control treatment. All the culture flasks were maintained at 30°C with aeration, and samples (2 mL) were acquired every two hours. The cell density of *R. solanacearum* was detected using a Nicolet Evolution 300 UV–Vis spectrometer at room temperature. Each set of experiments was repeated three times to calculate an average value.

### Adsorption on the Cell Surface

To further explore the mechanism of the cellular interaction between the two MgO powders and *R. solanacearum* cells, the cell morphology was investigated by SEM (S-3400N, Hitachi, Japan) and TEM (JEM-2100, JEOL, Japan). The cellular adsorption assays were further performed according to the method described in a previous study (Hartmann et al., 2010; Chen et al., 2014).

Briefly, the diluted *R. solanacearum* culture was maintained in an incubator overnight at 30°C with constant agitation to reach the 10^9^ concentration (OD_600_ = 1.0). Then, the bacterial suspensions were transferred into centrifuge tubes for centrifugation at 5000 × *g* for 4 min to remove the medium residue. Next, the bacteria were mixed with MgONPs or bulk MgO to obtain final dosages of 250 μg/mL. The cultures treated using only deionized water were considered as control groups. All the treated mixture were incubated for 4 h at 30°C and 200 g value, and the treated bacteria were gently washed with sterilized water three times and fixed with glutaraldehyde (2.5%) in sterilized water for another 1 h. A series of ethanol concentrations (30–100%) was used to dehydrate the samples until the bacteria were completely dry, and they were subsequently sputter-coated with gold. Finally, the morphological changes of the bacteria were then analyzed using SEM and TEM, and the elements were detected using energy dispersive X-ray spectroscopy (EDS) (Hitachi, Japan).

### Biofilm Formation Suppression Assay

The biofilm formation of *R. solanacearum* was performed according to a previous study with minor modifications (Zhang et al., 2014). Briefly, using crystal violet in 96-well polystyrene microtiter plates, 1 μL of the fresh bacterial suspension (OD_600_ = 1.0) was added to 199 μL of the B medium with MgONPs or bulk MgO added to guarantee a final concentration of 0, 50, 100, 150, 200, or 250 μg/mL, and the suspension was incubated for different times (24, 48, and 72 h) at 30°C without shaking to form a biofilm. After the supernatant was discarded, crystal violet was added into the wells for staining. Ethanol (95%) was used to adsorb the crystal violet from the biofilm. The experiments were conducted while measuring the absorbance at 488 nm. Each experiment was repeated a minimum of three times.

### Swimming and Twitching Motility Assay

The motility of bacteria is associated with several known virulence factors. Thus, the swimming and twitching motilities were determined in divided plates (Tahir et al., 2017) to detect the motility activity of the *R. solanacearum* exposed to the MgONPs and bulk MgO. Fresh *R. solanacearum* cells were cultured in the incubator overnight (OD_600_ = 1.0). Then, 2 μL of the nanoformulations were inoculated onto the different CPG agar media (Addy et al., 2012) containing various concentrations of MgO powders and incubated at 30°C. The CPG agar medium containing 0.3% (w/v) agar was used to determine the swimming motility, and 1.6% (w/v) agar (Raza et al., 2016) was used for the twitching motility determination. Later, after 3 days of incubation, the zones of *R. solanacearum* cell migration were measured. Meanwhile, colony-forming edges were observed after incubation for 2 days under a microscope. The experiments were replicated minimum of three times.

### LIVE/DEAD Assays to Infer the Cell Membrane Integrity

To determine and verify the membrane damage of the bacterial cells, a LIVE/DEAD BacLight Bacterial Viability Kit (Molecular Probes, Eugene, OR, United States) was used as the identification tool. The results of the LIVE/DEAD assays were indicated by the fluorescence intensity (red or green) of the stained microbial cells at a certain wavelength (Radzig et al., 2013). Specifically, *R. solanacearum* was cultivated in B medium overnight at 30°C without shaking. Afterward, the supernatant was removed, and fresh B medium blended with MgONPs (250 μg/mL) or bulk MgO (250 μg/mL) was added to the culture and incubated for 4 h. The staining reagent mixture (SYTO 9 and PI) was added to the bacterial suspension (washed in sterile water) and incubated in the dark for 15 min at room temperature. Next, the bacteria were observed by scanning confocal laser microscopy (Leica SP8, Germany) at a 488 nm wavelength for 0, 2, 4, 8, and 12 h.

### Flow Cytometry Observations

A flow cytometer was used to measure the changes in the light scattering of the cells after incubation with the nanoformulations (Kumar et al., 2011a). To assess the effect of the MgONPs on the *R. solanacearum* cells, the bacterial culture supernatant was removed after centrifugation at 5000 × *g* for 4 min and resuspended in sterilized water. The cells (10^6^ cfu/mL) collected at a logarithmic phase of growth were treated by MgONPs for 4 h and immediately stained using propidium iodide (PI) in the dark for 30 min. Next, the apoptosis in *R. solanacearum* was detected using flow cytometry (FACSAria, BD Biosciences, United States).

### Efflux of the Cytoplasmic Materials

Every normal bacterium has a complete cell membrane. Once the cell membrane is damaged, substances (DNA and RNA) in the cell are released, and the substances can be detected by a UV–vis spectrophotometer under 260 nm UV light (Chen et al., 2013). The bacteria suspension with MgONPs and bulk MgO (concentrations ranging from 0 to 250 μg/mL) was incubated for 4 h at 30°C. Then, the mixture was filtered to remove the bacteria, MgONPs and bulk MgO using a 0.22 μm drainage pin type filter. Finally, the OD value was monitored under 260 nm ultraviolet light.

### Determination of the Relative Reactive Oxygen Species (ROS)

Dichlorofluorescein diacetate (DCFH-DA) could enter the bacterial cells and further react with ROS to form the fluorescent compound dichlorofluorescein (DCF), which was detected by fluorescence at a wavelength of 488 nm (Applerot et al., 2012). Herein, DCFH-DA was used as a detection reagent to measure the intracellular ROS. The bacteria were cultivated overnight until the OD_600_ reached 1, and then the culture (1 mL) was centrifuged at 5000 × *g* for 4 min to divide the samples into three groups. Specific ROS scavenger rotenone (Sigma-Aldrich, United States) acting as negative control, which was conducted as previously described (Li F. et al., 2015). Bacterial cells were exposed to 250 μg/mL MgONPs suspension, 250 μg/mL MgONPs along with rotenone and sterilized water (blank control) for 4 h, respectively. Subsequently, these groups of suspension were gently washed with sterilized water three times and were added to 10 μM DCFH-DA at 30°C for 30 min in the dark. Finally, the fluorescence was determined using scanning confocal laser microscopy (Leica SP8, Germany) after washing the sample thoroughly with sterilized water. The experiments were repeated a minimum of three times.

### DNA Damage

The DNA damage of the *R. solanacearum* cells incubated with the nanomaterials was investigated using agarose gels, as previously described (Kumar et al., 2011a). The intensity of the DNA ladder was used to investigate the DNA fragmentation, and the following procedures were carried out. Bacterial cells were grown in the B medium at 30°C overnight, and the cells were prepared at the required concentration and transferred into 50 mL of the medium containing MgONPs with final concentrations of 0, 50, 100, 150, 200, and 250 μg/mL. A control sample was treated with DI water. After treatment for 4 h, DNA was extracted from all the bacteria by using a TIANamp Bacteria DNA Kit (Tiangen, China), and then an equal amount of DNA was stained with ethidium bromide for electrophoresis in a 1% agarose gel.

### Controlling Bacterial Wilt Using MgONPs and Bulk MgO in a Plant Growth Chamber

The virulence and pathogenicity of *R. solanacearum* to tobacco were examined *in vivo* using pot experiments (Tahir et al., 2017). The pathogenicity assay was conducted with tobacco plants (the cultivar “Yunyan 87”) at the four-leaf-old stage of growth in a matrix potting medium (Pindstrup Mosebrug A/S, Denmark). Consistent tobacco plants were selected, and 20 plants were prepared for each treatment. Here, the tobacco plants were inoculated with 15 mL of the *R. solanacearum* cell suspension (10^7^ cfu/mL) by irrigation around the basal part of the stem, and plants treated with DI water were used as control samples. Followed by cultivation for 24 h in a plant growth chamber under a temperature of 27 ± 1°C, a relative humidity of 85–90% and a light period of 14 h, the plants were drenched and treated with 50 mL of the MgONPs or bulk MgO suspension. Importantly, to reduce occasional errors, all the trials were established in a randomized complete block design. The experiments were carried out under the greenhouse conditions mentioned above. The disease severity was assessed for 21 days based on the proportion of wilted leaves; the detailed formula is as follows.

Disease index (%)=[Σ(ni×vi)÷(V×N)]×100,

where *ni* is the number of plants with the respective disease levels, *vi* is the disease level, *V* is the highest disease level (4), and *N* is the number of test plants per treatment. The disease levels were as follows: 0 – no wilt, 1 – one leaf wilted, 2 – two leaves wilted, 3 – four leaves wilted, and 4 – all leaves wilted.

### Toxicity of MgONPs Toward Tobacco Plant in a Growth Chamber

Plant growth was used to test the side effects of the nanoparticles (Raliya et al., 2015). Therefore, different concentrations (0, 50, 150, and 250 μg/mL) of MgONPs were applied to the tobacco at the four-leaf-old stage in matrix potting mediums by root exposure. During the experiments on the tobacco plants, 20 plants were tested in each treatment in a plant growth chamber (the environmental conditions were as detailed in the pathogenicity assay section). Finally, the physiological parameters of plant height and dry biomass were recorded on the 30th day after inoculation with MgONPs.

## Results and Discussion

### Nanoparticle Characterization Analysis

**FIGURE 1:**
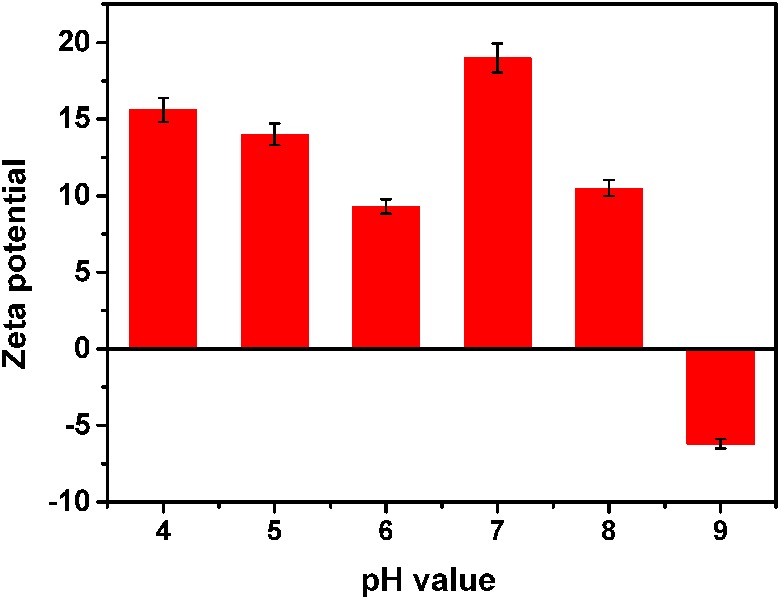
Zeta potential of the MgONPs dispersed in DI water. The pH values range from 4.0 to 9.0.

**FIGURE 2:**
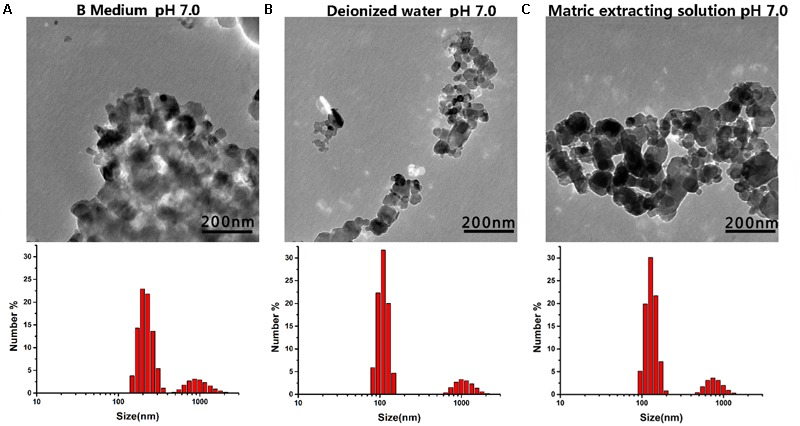
TEM and DLS images of the MgONPs dispersed in various solutions. **(A)** MgONPs suspended in the B Medium, **(B)** in the B Medium with DI water, and **(C)** in the matrix extraction solution.

The structure of the nanomaterials was related to their antibacterial activity. Moreover, the true size of the particles in the suspension was significantly different than the advertised size of the starting powders; this phenomenon has been reported by other researchers (Hristovski et al., 2005). Herein, we analyzed the representative chemical and physical properties of the MgONPs used in this study. First, SEM and TEM micrographs were obtained to characterize the morphology of the nanomaterials. As shown in Supplementary Figure S1 in the supporting information, the MgO microspheres exhibited of irregular but spherical particle-like shapes with porous organization coupled with partial agglomeration, and the diameter of the MgONPs was roughly in the range of 50–100 nm. The electrostatic interaction was measured by the zeta potential, which was obtained using a Malvern laser particle size analyzer (Malvern, United Kingdom), as shown in **Figure 1**. The mean zeta potential of the MgONPs exhibited a positive charge in the pH range from 4 to 8. When the powder was dispersed in a solution at pH 9, the nanoparticles exhibited a negative surface charge based on the electrostatic interactions between the positive charges on the surface and the ions in the solution. Subsequently, in addition to SEM (Supplementary Figure S1), dynamic light scattering (DLS) and TEM were also used to analyze the particle size distribution and stability of the MgONPs in different suspensions. For deionized water and the matrix extracting solution, the MgONPs appeared to contain similar particle size distributions, as shown in **Figures 2B,C**. Conversely, most of the MgONPs were assembled together in the B medium. The saline solution exhibited concentrated biomolecules, molecular oxygen, and ions, which were attributed to strong adhesion forces between the particles, causing aggregation. Moreover, an analogous investigation indicated that electrostatic attraction between the particles was an effect of coagulation (Zheng et al., 2018).

### Antibacterial Activity of the MgONPs Against *R. solanacearum In Vitro*

To determine whether MgONPs could inhibit the growth of or kill the plant pathogenic bacteria *R. solanacearum*, the MIC and MBC were measured. It was important to identify the effective concentration *in vitro*, as the recommended dosage of the nanomaterials must be determined to assess the potential for using these materials for crop protection in agriculture. As observed in Supplementary Table S1, the MIC and MBC of the MgONPs were 200 and 250 μg/mL, at which concentrations nearly all the *R. solanacearum* cells were killed, whereas the corresponding doses of the bulk MgO were 500 and 600 μg/mL, and the thiodiazole copper doses were 125 and 200 μg/mL, respectively. Although the three agents exhibited strong inhibition of the surviving germs at a specific concentration, the MgONPs and thiodiazole copper could curb or kill all the cells at a lower concentration, revealing that the nanomaterial had stronger antibacterial activity than the bulk material, even their antibacterial effect could match chemical agents.

Further, we compared the results with those of some chemical agents currently used against *R. solanacearum* according to the reference data (**Table 1**). The results showed that MgONPs could completely kill the bacteria at the concentration of 250 μg/mL, which is a bit higher than the corresponding concentration of thiodiazole copper. However, for the currently used chemical bactericides, antibacterial activity was tested with different methods, including the MIC, half maximal inhibitory concentration (IC_50_) or concentration for 50% of maximal effect (EC_50_). In **Table 1**, the MIC values of essential oil and streptomycin sulfate are much lower than that of MgONPs. However, streptomycin sulfate has been recognized to aggregate in the human body through the food chain and cause risks (Gu et al., 2018). The EC_50_ values of chloramphenicol and bismerthiazol was 20 and 59.69 μg/mL, respectively. Compared with chloramphenicol and bismerthiazol, the antimicrobial doses of MgONPs are slightly higher. However, further tests of these pesticides confirmed that the lethal concentration of MgONPs can match that of copper hydroxide and is even lower than those of coumarin and umbelliferone. More importantly, all the antibacterials could kill the pathogenic microorganisms, but with the advantages of not being genotoxic or cytotoxic to humans, MgONPs have great potential for controlling plant disease in the future.

**Table 1:** Several chemical pesticides against *R. solanacearum.*

Chemical	Effective	Method	Reference
Pesticides	Concentration		
	(μg/mL)		
MgONPs	250 μg/mL	MBC	in this experiment
	200 μg/mL	MIC	in this experiment
Thiodiazole copper	200 μg/mL	MBC	in this experiment
	125 μg/mL	MIC	in this experiment
Bismerthiazol	59.69 μg/mL	EC50	Li P. et al., 2015
Essential oil	12.5 μL	MIC	Purnavab et al., 2015
Chloramphenicol	20 μg/mL	IC50	Shiva Kumar et al., 2011
Streptomycin sulfate	3.91 μg/mL	MIC	Wattana-Amorn et al., 2016
Copper hydroxide	200 μg/mL	MIC	Lee et al., 2012

**FIGURE 3:**
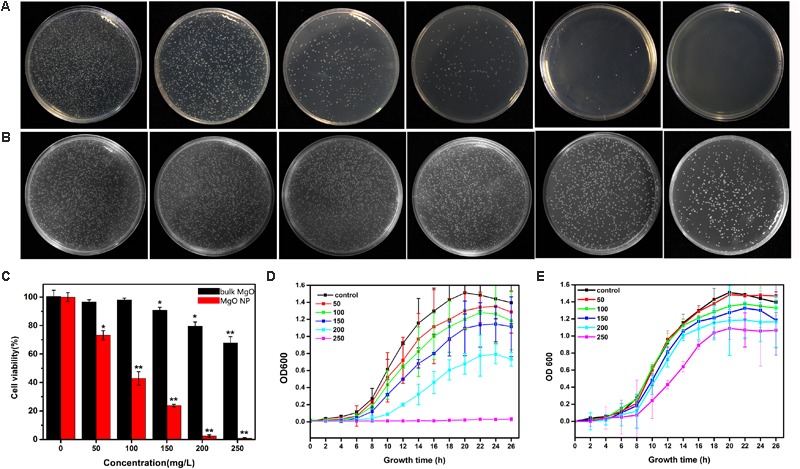
Antifungal activity of the MgONPs and bulk MgO against *R. solanacearum*. The bacterial colony images of *R. solanacearum* after the MgONP **(A)** or bulk MgO **(B)** treatment with different concentrations. The concentrations in both groups of agar plates from left to right are, successively, 0, 50, 100, 150, 200, and 250 μg/mL. The *R. solanacearum* cell viability rate **(C)** and growth curve after exposure to different concentrations of MgONPs **(D)** and bulk MgO **(E)**. These NA plates were cultivated for 3 days at 30°C without shaking. The error bars in the histograms represent the standard deviation, and ^∗^ and ^∗∗^ indicate *p* < 0.05 and *p* < 0.01, respectively.

Considering a more intuitive approach to comparing the efficiencies of the toxicities of the MgONPs, bulk MgO against *R. solanacearum*, the bacterial growth was further characterized by monitoring the viable cell counts using the classic colony counting method (Cui et al., 2016). The same number of *R. solanacearum* bacteria was inoculated on the CPG agar plates containing various concentrations of MgONPs and bulk MgO (0, 50, 100, 150, 200, and 250 mg/L), the respective cell viability rates were investigated by counting the CFU ratios of the respective treatments to the control. Agar plates without drugs were used as the blank groups, and corresponding photographs of the *R. solanacearum* colonies formed were recorded (**Figures 3A,B**). Notably, after interaction with the MgONPs and bulk MgO, the formation of bacterial colonies on the plates was obviously concentration dependent compared to the control (**Figures 3A,B**). It is worth noting that the MgONPs displayed more efficient toxicity to the *R. solanacearum* cells than the bulk MgO. In particular, the MgONPs exhibited a significantly lethal effect on bacteria at a concentration of 250 mg/L. As shown in **Figure 3C**, the survival rate remarkably only reached 0.80% compared to the untreated, nearly inducing complete death; this result was lower than that for the 250 mg/L bulk MgO (67.78%). These findings are consistent with previous studies regarding the toxicity of other nanoparticles toward phytopathogens, such as Ag, ZnO, and CuO (Dizaj et al., 2014).

The division of bacteria relies on binary fission, the most common mode of propagation in bacteria, which leads to bacterial population growth dynamics that can be roughly divided into four phases: the lag, exponential, stationary, and decline phases (Pinho et al., 2013). When bacteria are exposed to an unfavorable natural environment or adverse stress, such as nutritional deficiency, the bacterial community growth during the lag phases is impeded, the population size decreases (or enters the pending exponential phase), and eventually, the death rate becomes substantial, giving rise to the decline phases in comparison with the bacteria grown under normal conditions (Rolfe et al., 2012). Furthermore, the growth curve of *R. solanacearum* was used to evaluate the antibacterial effect of the MgONPs and bulk MgO in the B medium. The antibacterial activity accumulated progressively with increasing concentration of the two agent materials. MgONPs ranging from 50 to 250 μg/mL significantly inhibited the growth of *R. solanacearum* (**Figures 3D,E**), whereas less inhibition was found for the same concentrations of bulk MgO. The obvious toxicity of the MgONPs against the bacteria was observed at the lethal concentration (250 μg/mL). At this concentration, the growth curve results indicated that there was almost no bacterial growth, proving that the bacteria were killed. However, treatment with bulk MgO at the same concentration revealed weaker antibacterial activity. The results demonstrated that the MgONPs could kill bacterial cells at low concentrations. This difference may have resulted from the fact that the nanoparticles possess much higher BET surface areas and are pore volumes and smaller in size than the bulk MgO (Jeevanandam and Klabunde, 2002). Additionally, for the MgONPs, although agglomeration was a greater problem in the B medium than in water (**Figure 2**), few of the aggregates were larger than 100 nm. More importantly, the corresponding investigation above has proven that the lack of large aggregates likely plays a significant role in the inhibition of *R. solanacearum*.

Notably, these inhibitory effects were exhibited most clearly in the logarithmic growth phase, perhaps because the massively produced young bacteria at this stage are the most sensitive. Similarly, researchers recently found that the susceptibility of Gram-negative *E. coli* and *Pseudomonas aeruginosa* to graphene oxide is the highest in the exponential growth phase, in which the bacteria exhibit physiological changes, and bacteria are quite resistant during the stationary phase, in which the cells do not grow (Das et al., 2011). As inorganic antimicrobial agents, MgONPs have directly exhibited a wide spectrum of toxicity against several types of pathogens, such as Gram-negative (*E. coli*) and Gram-positive (*S. aureus*) bacteria (Cai et al., 2017) and even the fungi *Aspergillus niger* and *Penicillium oxalicum* (Sierra-Fernandez et al., 2017); however, there is a lack of research on the detailed mechanism. In summary, the results of the above experiments confirmed that the MgONPs exhibited predominant antimicrobial properties in the presence of *R. solanacearum.*

### Morphological Damage Observed by SEM and TEM

The SEM is typically applied to observe external cellular morphological changes, and TEM can be used in combination and applied to bacterial sections to explore the ultrastructural changes in pathogenic cells (Sun et al., 2017). We used both SEM and TEM to investigate the interaction between the two forms of MgO and the bacterial cells to compare the cellular changes of the untreated and treated cells during the exponential growth phases.

**FIGURE 4:**
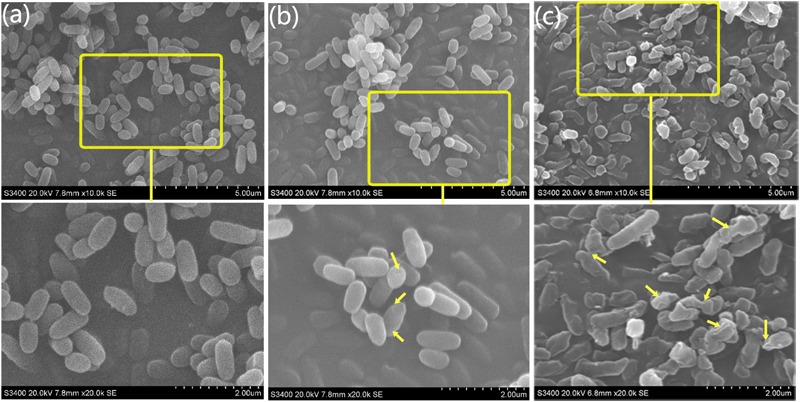
SEM micrographs of *R. solanacearum* using scanning electron microscopy (SEM). The round and intact cells are the control cells. **(a)** After treatment with 250 μg/mL bulk MgO, small holes can be seen in **(b)**, while the cells incubated with 250 μg/mL MgONPs exhibited deep craters and burst cells. **(c)** The lower screens are the enlarged parts of the upper yellow rectangles.

The integrity of the morphology was maintained in the untreated group (**Figure 4a**). In contrast, the colonies of bacteria exposed to the MgONPs (250 μg/mL) were severely disrupted, exhibiting shrinkage (the yellow arrows in **Figure 4c**). However, for the bacteria treated with bulk MgO, only small sections of the cell membrane became rough (the yellow arrows in **Figure 4b**); portions of hollow and distorted cell structures were observed. As observed in the TEM images (**Figure 6**) and indicated by the hollows cells (red arrows), the MgONPs could destroy or disintegrate cell walls to penetrate the bacterial cells; this damage could eventually lead to leakage of the intracellular content, resulting in cell death (**Figures 6d,e**), which is consistent with the results of a previous study (Jin and He, 2011).

**FIGURE 5:**
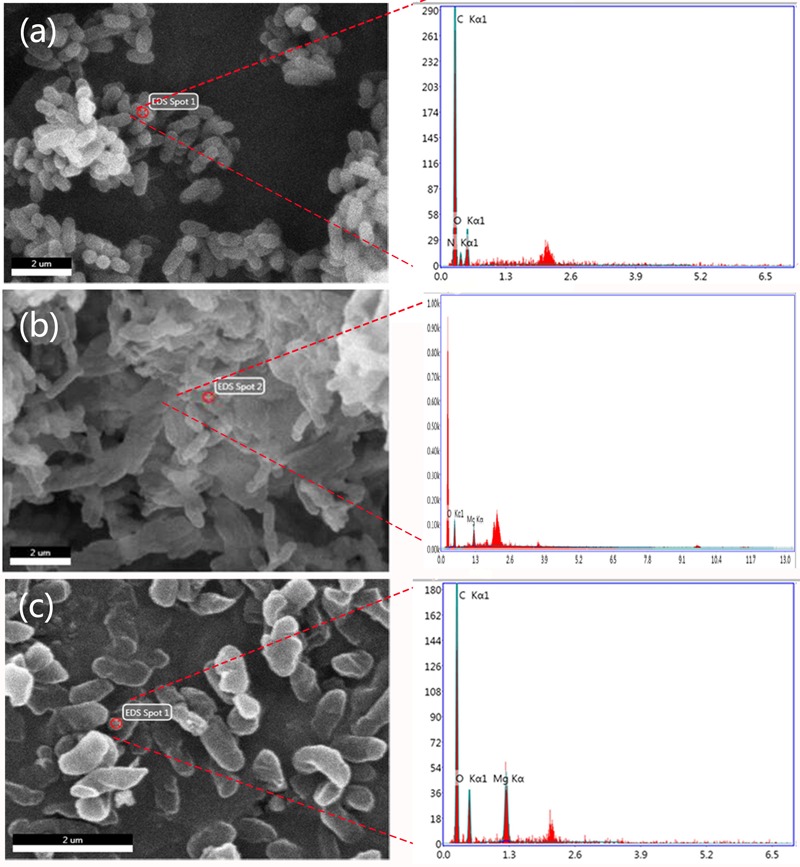
Scanning electron microscopy images with energy dispersive X-ray spectroscopic (EDS) analysis of **(a)** *R. solanacearum*, **(b)** the cells treated with 250 μg/mL bulk MgO, and **(c)** those treated with 250 μg/mL MgONPs.

**FIGURE 6:**
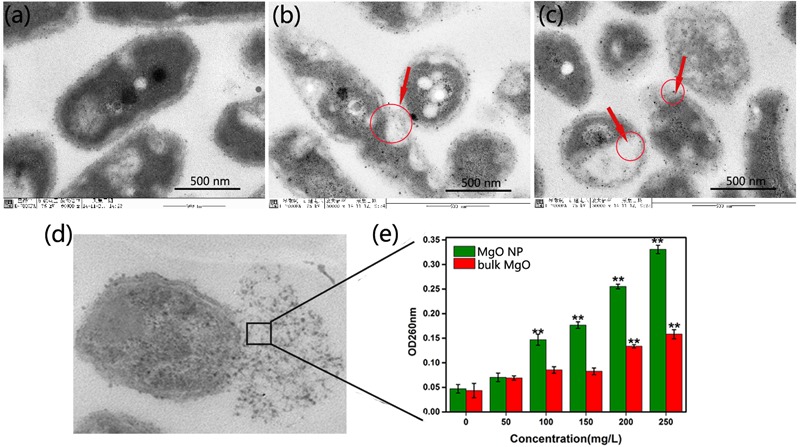
TEM images of *R. solanacearum* cells treated with DI water **(a)**, 250 μg/mL bulk MgO **(b)** and 250 μg/mL MgONPs **(c)**. The overnight cultured bacterial suspension was supplemented with different forms of MgO and incubated at 30°C for 2 h. **(d)** Observations of the damaged bacterial membranes of the MgONP-treated bacteria using TEM. **(e)** The absorbance of the efflux of the cytoplasmic materials (DNA and RNA) at 260 nm after the cells were incubated with different concentrations of bulk MgO and MgONPs. The error bars in the histograms represent the standard deviation, and ^∗∗^ indicate *p* < 0.01.

In addition, an energy dispersive spectrometer (EDS) with SEM analysis (**Figure 5c**) was used to determine the adsorption of MgONPs on the cell surfaces, and a similar phenomenon was observed under the bulk-MgO-treated conditions (**Figure 5b**). We speculated that both MgO powders adhered to the bacterial cell walls, and the expected results were obtained. Specifically, in the zoomed-in region, compared to the control cells with a plump without any Mg element (**Figure 5a**), glossy and rod-like morphological structure, after incubation with the MgONPs, significant abnormalities in the cell morphology and incomplete envelopes were observed (**Figure 4c**). However, at a high concentration (250 μg/mL) of bulk MgO, there were no apparent abnormalities in the surface morphologies of most cells (**Figure 4b**), whereas there was still an Mg element evident on the cell surface (**Figure 5b**). The obvious enhanced antibacterial activity of the MgONPs has attracted our attention for determining their antimicrobial mechanism. Regarding the adsorption and surface reaction center, the surface of MgO contains a large number of active sites, such as Mg^2+^, O^-^, Lewis acids, limited hydroxyl groups, free hydroxyl groups, and cationic and anionic holes (Jeevanandam and Klabunde, 2002), which may be beneficial to its antibacterial activity. Previous studies proved that with decreasing dimension of powder particles, the surface molecule are fraction, which in turn improves some properties, such as the dissolution rate and catalytic activity (Dizaj et al., 2014). Moreover, Sawai et al. (1996) revealed that MgONPs exhibited varying levels of toxicity depending on the different types of bacteria; better inhibition was observed for rod-shaped bacteria (such as *E. coli* and *Bacillus subtilis*) than for spherical-shaped bacteria. In this regard, MgONPs have potential applications as rod-shaped bactericides and can be remarkably applied to *R. solanacearum.* Moreover, to the best of our knowledge, as a type of Gram-negative bacteria, *R. solanacearum* is a negatively charged bacterial cell. Interestingly, it has been reported that electrostatic attraction between negatively charged cells and positively charged nanoparticles could play a vital role in the toxicity of nanoparticles toward microorganisms (Seil and Webster, 2012). The oppositely charged nanoparticles easily and tightly binding to the cellular surface, thus inducing aggregation and then increasing the toxicity toward the bacteria (Le Ouay and Stellacci, 2015). Though the interactions of nanoparticles with *R. solanacearum* cells are not meant to alter the subsequent cell functions, the tremendous damage to the cell wall is inspiring, because the abnormalities of *R. solanacearum* are indicative of the primary antibacterial mechanism of nanoparticles. Hence, these interactions are possibly one crucial pathway to penetrating bacteria cells to eventually kill the bacteria.

### Bacterial Cytomembrane Integrity Measurement

There is a strong cell envelope that encapsulates Gram-negative bacterial cells. The cell envelope, which is composed of the polymer peptidoglycan, determines the cell shape and protects it from osmotic lysis (Bindhu et al., 2016). Furthermore, the oxygen vacancies of MgONPs are located at the surface of the cell or near the particle surface (Dizaj et al., 2014). Therefore, it is essential to further determine whether the antibacterial behavior of MgONPs can be attributed to direct injury of the cytoplasmic membrane.

We conducted experiments using a LIVE/DEAD BacLight Bacterial Viability Kit, which was a convenient and high-efficiency method for monitoring the membrane integrity of a cell. Fluorescent dyes have specific intracellular binding sites that can be utilized to reveal important physiologic information of the bacteria (Sun et al., 2017). After staining with the LIVE/DEAD kit for a short time, cells with a compromised membrane that are considered to be dead or dying will stain red, whereas cells with an intact membrane will stain green. **Figures 8d,e** show the fluorescence images of *R. solanacearum* in the presence of MgONPs or bulk MgO for 2, 4, 8, and 12 h. In the control images, all of the cells were alive because they were stained green. However, after incubation with 250 mg/mL MgONPs or bulk MgO, the images increasingly presented red fluorescence as the incubation time increased. Moreover, there were a large number of dead cells and few living cells in the images of the MgONP-treated samples. From the results, we can conclude that the antibacterial activity of the MgONPs and bulk MgO occurred in a dose- and time-dependent manner, and these results further confirmed that a higher inhibition was induced by the nanoparticles, which agrees well with previously published results on carbon-based nanomaterials (Chen et al., 2016). The efficiency of graphene and CNTs for inactivating *E. coli* and *B. subtilis* bacteria increased with increasing shaking speed, time, and concentration (Liu et al., 2010, 2011). Actually, large quantities of engineered nanoparticles (ENPs) have been proven to display these effects as well, such as ZnO, CuO, and AgNPs (Ivask et al., 2014). Moreover, a previous study showed that silver-nanoparticle-decorated quercetin nanoparticles indeed killed bacterial cells, rather than only harming the cells (Sun et al., 2017), but MgONPs have been comparatively less researched.

**FIGURE 7:**
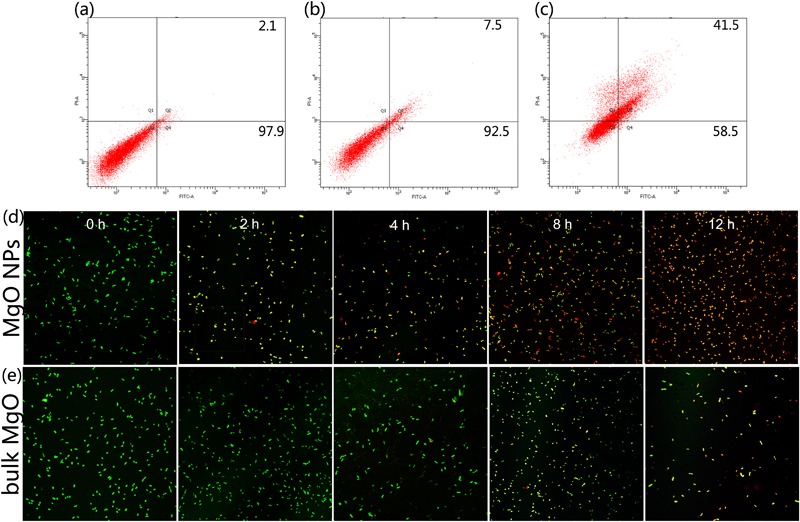
Flow cytometry images of *R. solanacearum* cells after incubation with **(a)** sterile water, **(b)** bulk MgO, and **(c)** MgONPs. Confocal fluorescence microscopic images were obtained using the LIVE/DEAD BacLight Bacterial Viability and Counting Kit for different times (0, 2, 4, 8, and 12 h) of treatment with **(d)** MgONPs and **(e)** bulk MgO. Cells with green fluorescence represent live bacteria, whereas red cells are representative of dead bacteria.

Flow cytometry usually allows for the rapid detection of the internalization of nanoparticles in live bacteria (Kumar et al., 2011a). In addition, *E. coli* exhibited a high degree of cell membrane depolarization and a large amount of leakage during nanoparticle treatment, as determined from a flow cytometry analysis (Kumar et al., 2011b). PI is a type of fluorochrome that is incapable of passing through intact cell membranes but can pass through cells that are injured or dead. After entering into cells, the stained double-stranded DNA was observed using ultraviolet light. Herein, according to the flow cytometric analysis in combination with PI, we obtained a rapid and precise evaluation of the cellular structural damage. As demonstrated in **Figure 7c**, the apoptotic cell ratio of *R. solanacearum* increased to 41.5% in comparison with the control (2.1%) (**Figure 7a**) when incubated with 250 μg/mL MgONPs. However, bulk MgO with the same dosage only attained an apoptotic cell ratio of 7.5% (**Figure 7b**). Collectively, the data presence of that the presented MgONPs increased the membrane permeability of the *R. solanacearum* cells, possibly resulting in cell injury or death.

In this assay, the cells were treated with nanomaterials in B medium *in vitro*, in which the MgONPs reflected different solubilities (**Figure 2**). This result is not surprising, as it is known that metal nanoparticles remain dispersed under different solution conditions. Additionally, a detailed analysis distinguished the inhibitory effects toward *R. solanacearum*. The MgONPs showed similar particle size distributions in deionized water and a matrix extraction solution. In contrast, more particles assembled in the B medium containing a saline solution. Analogously, previous reports have disclosed that in KNO_3_^-^ rich media, the interactions between surface organic materials and NPs caused AgNPs to agglomerate more easily than in distilled water (Zook et al., 2012). Overall, though some aggregation occurred in the B medium, the MgONPs still displayed strong antimicrobial effects. It seems that this behavior had little or no impact on the toxicity action against the pathogens.

Meanwhile, bacterial leakage could definitely identify whether a cell membrane was damaged by MgONPs or bulk MgO because, once cell membranes are affected by external factors, large molecules, such as DNA and RNA will leach out and rapidly increase the absorbance at 260 nm when using UV–vis spectroscopy. **Figure 6e** showed that the two groups of MgO A_260_ increased in a dose-dependent manner. The A_260_ value indicated that the membranes were severely destroyed, especially in the presence of 250 μg/mL MgONPs. In this case, the cytoplasm leakage sharply increased, as indicated in **Figure 7d** compared with **Figure 7e**, with significant membrane permeabilization. Jin and He (2011) also observed a variety of cellular injuries after introducing MgONPs to *E. coli* and *Salmonella Stanley*, proving the deformation and damage of the cell membrane, which further resulted in the leakage of cell inclusions and, eventually, death.

### Suppressing the Formation of the Biofilm and Motility

Similar to other pathogenic bacteria, *R. solanacearum* attaches to surfaces and aggregates to form biofilms. Biofilms are bacterial cell communities wrapped in a self-produced hydrated polymeric matrix that adheres to a living surface (Zhang et al., 2014). Importantly, the formation of biofilms is a new model system, and these biofilms probably protect the bacteria from immune attacks by the host and may contribute to bacterial survival during latent infections and saprophytic life (Bridier et al., 2011). Hence, to some extent, the loss of biofilm formation ability by the bacteria may indicate less virulence when invading and colonizing the host tissue directly. Therefore, in an attempt to investigate the mechanism by which MgONPs activated *R. solanacearum*, we further proposed to determine if the biofilm formation was inhibited.

As illustrated in Supplementary Figures S2a,b, the MgONPs significantly reduced the biofilm growth, and the biofilm formation gradually decreased with the bulk MgO treatments. In both cases, similar to the viability tests, the inhibitory efficiency tended to become stronger as the concentration increased. Interestingly, the biofilms suddenly decreased after incubation with the MgONPs for 24 h, but the inhibition effects of the bulk MgO were moderate. However, exposure to low concentration treatments (50, 100, and 150 μg/mL) of MgONPs for 48 and 72 h of incubation did not result in significant mobility changes in the bacterial cells. Only the 200 and 250 μg/mL treatments exhibited high inhibitory effects on the *R. solanacearum* biofilm formation when the cells were exposed to nanoparticles for 48 and 72 h, respectively. The biofilm formation was reduced by 60.7 and 71.2% after 24 h and by 67.1 and 72.3% after 72 h, respectively, compared with the control.

Inspired by a previous study, for pathogenic bacteria, surface attachment is typically the initial step in pathogenesis and aggregation, especially for *R. solanacearum* (Addy et al., 2012). Our results indicated that the MgONPs restrained the biofilm formation, implying that they not only prevented the infection process of *R. solanacearum* on the host plant but also improved the bacterial susceptibility to antibiotics. Hence, the nanoparticles have potential applications as highly efficient agriculture antimicrobial agents. Moreover, a similar antibacterial mechanism was also proposed by other researchers who were investigating the toxicity of metals and metal nanoparticles against bacteria (Radzig et al., 2013). Radzig et al. (2013) found that the viability of *E. coli* AB1157 cells in biofilms was considerably reduced by AgNPs at concentrations between 100 and 150 μg/mL.

**FIGURE 8:**
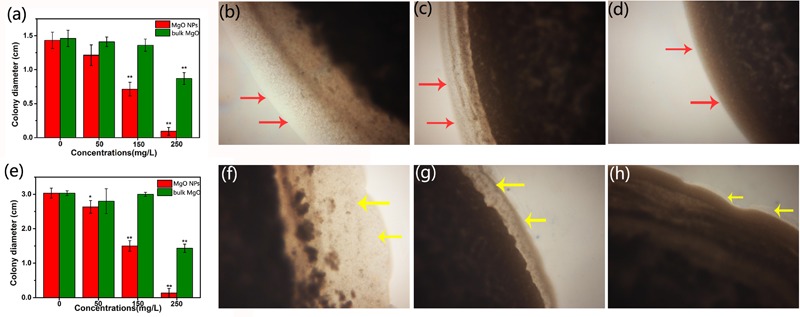
Effect of the MgONPs and bulk MgO on the motility traits of *R. solanacearum*. The images are the twitching motility **(a)** and swimming motility **(e)** of *R. solanacearum* after incubation with various concentrations of MgONPs and bulk MgO. The left side representative microscope images show some details, the twitching motility **(b)** of the untreated control, **(c)** with 200 μg/mL bulk MgO, and **(d)** with 200 μg/mL MgONPs and the swarming motility **(f)** of the untreated control, **(g)** with 200 μg/mL bulk MgO, and **(h)** with 200 μg/mL MgONPs. The arrows indicate the wider peripheral colony fringe of *R. solanacearum*. The error bars in the histograms represent the standard deviation, and ^∗^ and ^∗∗^ indicate *p* < 0.05 and *p* < 0.01, respectively.

In addition, a combined study of MgONPs and their motility activity provided a unique perspective for understanding how MgONPs influence biofilm formation. Surprisingly, there were significant reductions in both the swimming and the twitching motilities of the bacteria when inoculated onto the agar medium containing nanoparticles. As expected, the inhibition rates of the motility by the MgONPs were 95.60 and 93.40% for swimming and twitching, respectively, in comparison with the nonexposed control (**Figures 8a,e**). The peripheral colony fringe under the control conditions developed a relatively wide length and width, whereas the bacterial colony of the MgONP treatment was smooth. After exposure to a 250 μg/mL dosage, the twitching motility of *R. solanacearum* was limited to 0.93 mm in the presence of the MgONPs and 8.80 mm in the presence of MgO. In contrast, that of the control was 14.10 mm. Representative microscope images under 200 μg/mL are visualized in **Figures 8b–d**. The swimming motility was restricted to 1.33 mm with the 250 μg/mL MgONPs, and it was restricted to 14.30 mm for the same concentration of bulk MgO compared to the control (30.20 mm) (**Figure 8e**). The typical inhibitory effects observed in this experiment are shown in **Figures 8f–h**. Altogether, these results provided insight into how the MgONPs affect these biological behaviors, which is of paramount importance for understanding the virulence of *R. solanacearum*, especially regarding the invasion and colonization of host plants. This phenomenon inferred that adverse conditions could exert an influence on the transcriptional expression of the motility-related genes of *R. solanacearum* (Tahir et al., 2017). However, it still remains insufficient to kill the cells because biofilms protect the bacteria inside from the action of a bactericide, although other studies have shown stable performance in controlling biofilm formation (Tahir et al., 2017).

### Intracellular Oxidative Stress Accumulation and DNA Damage Induced by MgONPs

Considering the stronger activity of MgONPs than the bulk MgO against *R. solanacearum*, as indicated in the above studies, we only used the MgONPs in the subsequent studies to investigate the further toxicity mechanisms. Oxygen is necessary for most living organisms, but it is also a precursor of ROS, which can damage cellular components such as proteins, lipids, and nucleic acids (Applerot et al., 2012). Many previous investigations on nanomaterial cytotoxicity focused on oxidative stress as a common pathway for the toxicity mechanism. Notably, the production of ROS likely leads to membrane damage because of lipid peroxidation, which causes cell material leakage and influences the respiratory activity to eventually cause cell death (Le Ouay and Stellacci, 2015). Moreover, in general, among the several paths of oxidative stress, one involves ROS-mediated oxidative stress, and the commonly accepted toxic effects exerted by nanomaterials involve the production of ROS (Horst et al., 2013).

**FIGURE 9:**
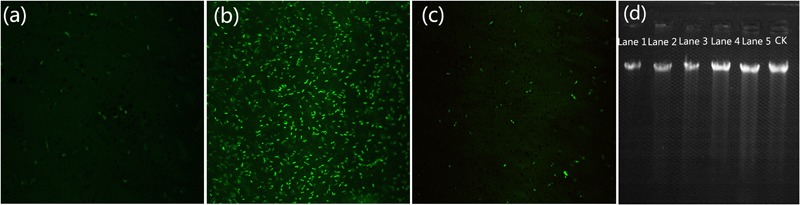
Formation of ROS in *R. solanacearum* cells after a 4 h incubation period without **(a)**, with **(b)** MgONPs only and with **(c)** rotenone before MgONPs treatment. ROS were detected by fluorescence measurements using the DCF indicator. **(d)** Electrophoresis analysis of genomic DNA in *R. solanacearum* cells treated with different concentrations of MgONPs for 4 h. Lanes 1, 2, 3, 4, and 5 represent treatments with 250, 200, 150, 100, and 50 μg/mL MgONPs, respectively.

As shown in **Figures 9a–c**, a significant increase in the DCF fluorescence intensity after treatment with 250 μg/mL MgONPs was observed in comparison with water only (blank control) and the ROS inhibitor rotenone (negative control). The mitochondrion is an important site to produce ROS. However, rotenone, a scavenger of the mitochondrial electron transport chains, could significantly decrease the ROS content (Li F. et al., 2015). Therefore, the fluorescence degree indicates MgONPs did contribute to ROS generation. The production of ROS increased the oxidative stress in the cells, which in turn induced DNA, protein, and cell damage (Le Ouay and Stellacci, 2015). The results indicated that the potent antibacterial activity of the MgONPs was probably related to ROS generation, which occurred on the surface of and in the bacterial cells. ROS should thus be perceived as a link to the antibacterial activity of these powders since these powerful oxidizing agents are known to be very detrimental to fully functional, metabolizing healthy cells (Le Ouay and Stellacci, 2015). For example, once O_2_^-^ free radicals were formed, consistent with the results of a previous study (Krishnamoorthy et al., 2012a), through the single-electron reduction reaction activity of O_2_^-^, the oxygen vacancy catalysis of MgO crystals could dissolve the oxygen in the water on the surface. O_2_^-^ has strong oxidizing properties and is capable of penetrating into cells, thus eventually killing the bacteria (Sirelkhatim et al., 2015). Similar mechanisms were also found in the apoptosis process of cancer cells under MgONP stress (Krishnamoorthy et al., 2012b).

In our study, the MgONPs directly adhered to the ambient cell wall of *R. solanacearum*, inducing a significant increase in the Mg element content (**Figure 5c**), and in the meantime, the nanoparticles generated intracellular and extracellular ROS, resulting in membrane and organelle damage (**Figures 4c, 6c**). Subsequently, this damage caused cytoplasm leakage, such as of protein and DNA, in the cells (**Figure 9**) and eventually led to cellular inactivation.

As expected, the dissolved MgONPs were positively charged in the neutral solution, enabling electrostatic interactions with the negatively charged bacteria. Indeed, a similar conclusion was obtained by a previous report (Liu et al., 2018). The *R. solanacearum* bacterial cells were combined with MgONPs by electrostatic forces. Meanwhile, Mg ions were released from the MgONPs and transported to the cytoplasm (Jin and He, 2011). Furthermore, portions of the MgONPs easily reacted with water to form Mg(OH)_2_, which could directly penetrate the cell wall, enter the cell, and then cause cell death (Dong et al., 2010). Many studies have focused on the Mg(OH)_2_ antibacterial mechanism (Dong et al., 2011; Pan et al., 2013). This focus is not surprising, as it is known that Mg(OH)_2_, as a type of alkali that is slightly soluble in water, can lose OH^-^ in water and increases the pH value. This behavior also has an impact on O_2_^-^, which is more stable in alkaline environments, and contributes to the higher antibacterial effect (Yao et al., 2018). Moreover, this fact illustrated that the action of the MgONPs as a strong bactericide agent was not just confined to one mechanism but involved complicated and multitudinous systems.

To test the direct damage of *R. solanacearum* DNA by the MgONPs, the isolated DNA extracted from the MgONP-treated bacterial cells was analyzed by agarose gel electrophoresis. The genomic DNA intensity bands can be observed in **Figure 9d**. It can be seen that the intensity of the genomic DNA band under the MgONPs treatment was lower than that of the control, exhibiting significant DNA fragmentation. In addition, as the concentration of the MgONPs increased, a significant decrease in the intensity of the genomic DNA band was observed. Analogously, other studies have evaluated the antibacterial mechanism of other metal powders (Zheng et al., 2018). In particular, previous studies have proven that nanoparticles can inhibit the synthesis of cell wall components, cellular proteins, DNA, and the RNA of bacteria (Raghupathi et al., 2011). In summary, the results demonstrated that the MgONPs could significantly improve the toxicity action of the Mg element via direct-contact interactions with *R. solanacearum*.

Though possessing weaker antimicrobial properties compared with the MgONPs, the slight abnormalities of the *R. solanacearum* cells and even the inhibition effect under bulk MgO treatment could not be ignored. As indicated by direct observations, some possible mechanisms have been proposed to describe cells suffering a variety of injuries to their microstructures from both physical and chemical damage. The injuries were probably due to not only the sharp edges of the MgO contacting the membranes but also the release of Mg^2+^, the dissolution of O_2_^-^, adsorption on the cells, and a great number of complex redox reactions. According to a previous review (Le Ouay and Stellacci, 2015), we speculated that the release of Mg^2+^ was likely a primary step of antimicrobial activity when in contact with bacteria. As such, as the size of the metals decrease, the biocidal property could be promoted by increasing the release of ions; for instance, nanometals exhibited a preponderant role in the antimicrobial efficacy (Zheng et al., 2018), but the mechanism is not the only one. Another crucial aspect is the shape and dissolution of metals, which have an influence on the antimicrobial effect (Misra et al., 2012). It should be noted that differences in the pore volumes of the two materials were tested for interaction with the microorganisms and were found to play a vital role in the antibacterial effects (Stark, 2011). In the cases, there is no doubt that these synergistic effects of the MgONPs were responsible for the remarkable antibacterial action toward *R. solanacearum* compared to the bulk MgO.

### Control Efficiency of the MgONPs and Bulk MgO on the Tobacco Bacterial Wilt Caused by *R. solanacearum In Vivo*

**FIGURE 10:**
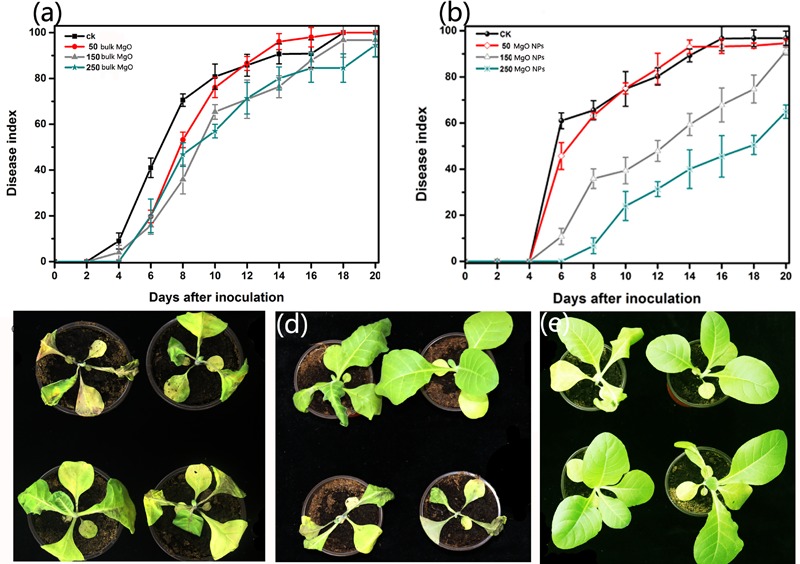
Antibacterial activity of the MgONPs and bulk MgO against *R. solanacearum in vivo*. The disease indexes of the tobacco plant were investigated after treatment with different concentrations (50, 100, 150, and 250 μg/mL) of **(a)** bulk MgO and **(b)** MgONPs. All the plants were tested in the set up in a randomized complete block design, and the error bars represents the SEM. The optical photographs 15 days after irrigation with **(c)** distilled water and **(d)** 250 mg/mL bulk MgO and **(e)** MgONPs were taken with a Nikon camera to survey the control efficiency.

To demonstrate that the MgONPs inhibited the bacterial wilt caused by *R. solanacearum*, pot experiments were conducted under greenhouse conditions *in vivo*. Tobacco plants were inoculated with *R. solanacearum* via noninjured root inoculation and the disease severity was calculated every 2 days. From **Figure 10**, it can be seen that the application of MgONPs in the greenhouse experiment remarkably reduced the destructiveness of the bacterial wilt disease compared to the untreated control. Treatment with 250 μg/mL MgONPs significantly reduced the disease severity, resulting in a 62.5% wilt index at 21 days (**Figure 10a**), whereas in the presence of an equal concentration of bulk MgO, a 93.20% wilt index was observed, compared with a 98.5% wilt index in the control (**Figure 10b**). The plant growth states were visualized according to optical photographs. The application of MgONPs could significantly reduce the severity of the bacterial wilt disease on tobacco, as shown in **Figure 10e**, and few flaccid leaves were observed when the plants were treated with 250 μg/mL MgONPs in contrast to the untreated plants, which displayed total plant wilting and death (**Figure 10c**). Even with the bulk MgO with a uniform concentration, the plants wilted and died (**Figure 10d**). However, the results indicate that adhesion of the MgONPs on the tobacco root surface occurred, and homologous phenomena have also been observed with other nanomaterials (Ocsoy et al., 2013). Even so, there was still a significant reduction in the disease severity after interaction with the MgONPs, which likely partly inhibited the virulence and pathogenicity of *R. solanacearum*. More importantly, MgONPs exhibited a variety of advantages, such as zero phytotoxicity, a low dose requirement and thermal stability combined with nongenotoxicity, as well as noncytotoxicity to humans, enabling brilliant application prospects for plant protection (Khot et al., 2012). It was previously observed that by using MgONPs at a concentration of up to 1%, the MgONPs significantly reduced the incidence of disease by inducing systemic resistance in tomato plants against *R. solanacearum* (Imada et al., 2016). Analogously, massive numbers of nanomaterials have been observed to control other phytopathogens in agricultural plant management. Graphene oxide nanomaterials showed high antimicrobial action toward phytopathogenic microorganisms when applied to the bacteria *Pseudomonas syringae* and *Xanthomonas campestris pv. undulosa* and even to two fungal pathogens (*F. graminearum* and *Fusarium oxysporum*) (Chen et al., 2014). Furthermore, Mg as an essential mineral element for plants health has been used for balanced nutrition and participates in a wide range of physiological functions (involved in the defense, virulence, or pathogenesis of plants). As an important contributor, the benefits of Mg can result in increasing the resistance of tissues to reduce disease, which is an underutilized tool for hindering the incidence of diseases (Huber and Jones, 2013). It has to be mentioned that MgONP suspensions could release several tens of thousands of Mg atoms, and in our following study, it was observed that MgONPs could be absorbed into plant tissue and provide nutrients that are favorable for plant growth (data unpublished); a similar effect was also proven in watermelon plants by Wang et al. (2013). Next, we will expand these investigations by exploring the effects of MgONPs on various plant species.

### Toxicity Test by Tobacco Plant in a Plant Growth Chamber

Nanotoxicity, as an emerging concept, is attracting increasing attention with the fast development of nanometer technology (Ruotolo et al., 2018). Since MgONPs were confirmed as an outstanding bactericide in our research, concern about the subsequent side effects of their use in plants and the potential adverse effects on the environment and human health needed to be investigated.

**FIGURE 11:**
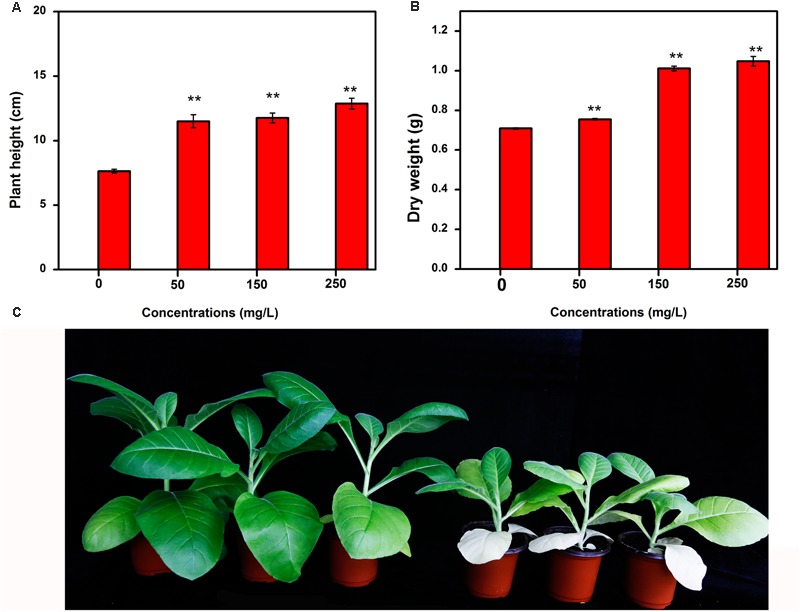
Effect of MgONPs on plant height and weight of tobacco after 30th day of treatment. **(A)** Plant height and **(B)** dry weight shows the growth after exposure to different concentrations of MgONPs. **(C)** Represents the tobacco plants growth treated with 250 μg/mL MgONPs (the 3 plants on the left) and with water (the 3 plants on the right), respectively. Error bar in the histograms represents the standard deviation, and ^∗^ and ^∗∗^ indicate *p* < 0.05 and *p* < 0.01, respectively.

In this study, tobacco was used to assess the toxicity and bioavailability of MgONPs. The tobacco was exposed to MgONPs with concentrations ranging from 0 to 250 μg/mL in a matrix potting medium for 30 days in a plant chamber. As shown in **Figure 11**, increased height and weight of tobacco plants was observed after being treated by MgONPs. In detail, the concentration of 50 μg/mL did not have a significant effect on the ground part weight. Additionally, the average plant height and weight under 150–250 μg/mL were highly comparable to those of the control, particularly for 250 μg/mL MgONPs (**Figure 11C**). Compared to the control, plants treated with 50, 150, and 250 μg/mL MgONPs showed the ground part height was 11.5, 11.77, and 12.87 cm (**Figure 11A**), respectively, with the corresponding increase of 50.72, 54.26, and 68.68%. Moreover, the dry weight in the ground part increased 47.77% with the treatment of 250 μg/mL MgONPs (**Figure 11B**) which is the best effect compared with the others. Therefore, it has been established that 0–250 μg/mL MgONPs could enhance tobacco plant growth.

A similar result was found by previous research (Rathore and Tarafdar, 2015), in which wheat achieved higher crop production with the application of MgONPs, which even showed the possibility of improving the native nutrient mobilization and soil health. Many reports also observed that different metal nanoscale particles enhanced plant growth (Tarafdar et al., 2014); for example, TiO_2_ nanoparticles as a photocatalytic bactericide could be incorporated into fertilizers to improve crop yield via nitrogen photoreduction (Raliya et al., 2015). In addition, some reports revealed that the nanoparticles increased the in hydro-mineral flow and nutrient uptake by creating new root pores (Castiglione et al., 2011) and even enhanced the chlorophyll content in the plants (Shah et al., 2015). Therefore, our study provided an indication that MgONPs in the range of concentrations from 0 to 250 μg/mL could be used as an effective way to protect plants, and in the meantime, they may improve agricultural production. The results may be further evaluated to guarantee the safe use of MgONPs in agriculture.

**FIGURE 12:**
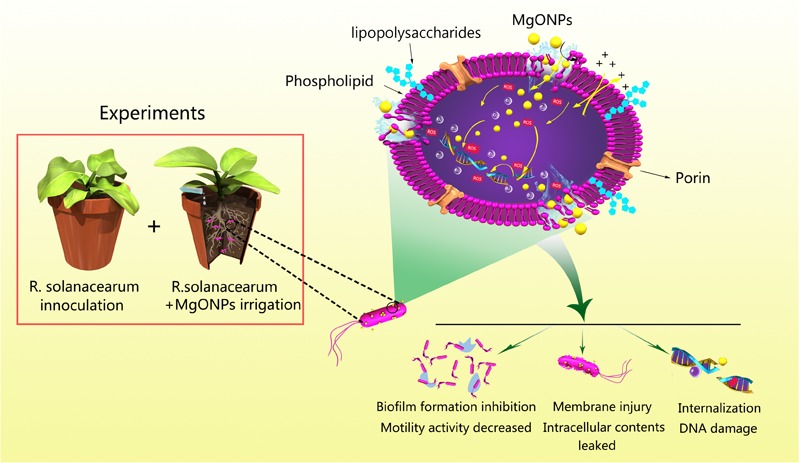
Schematic diagram of the interactions between the MgONPs and *R. solanacearum* and the toxicity mechanisms of the MgONPs. In this process, the bacterial cells were aggregated with the MgONPs. The interactions resulted in the destruction of the cell membrane, causing cytoplasm leakage and abnormal morphology and inhibiting the biofilm and motility activity. Additionally, the MgONPs induced the generation of ROS, and once the oxidative species entered into the cytoplasm, they damaged and fragmented the DNA, ultimately resulting in cell death.

Overall, MgONPs, which are potential antibacterial agents, are emerging as a class of pronounced therapeutics for bacterial wilt disease. According to **Figure 12**, there is a direct interaction mode between MgONPs and *R. solanacearum*, and multiple antibacterial mechanisms have been proposed. After the cells were exposed to the nanoparticle suspensions, the MgONPs could strongly perturb the essential functions of the cell membranes simultaneously, mainly because the positively charged MgONPs directly adhered to the negatively charged *R. solanacearum.* First, the MgONPs damaged the cell wall and membrane structure via mechanical injury or ROS production to initiate cytoplasm leakage in the cells. Then, the MgONPs inhibited biofilm aggregation on the surface and reduced the bacterial motility. In addition, after permeating into the cells, the ROS could destroy DNA, eventually resulting in bacterial death. Ultimately, the MgONPs remarkably reduced wild diseases compared to the untreated control.

## Conclusion

In this study, MgONPs exhibited superior antibacterial properties against *R. solanacearum* at an exceedingly low concentration (250 μg/mL) compared to bulk MgO, which could prevent diseases in the host plant. The antibacterial activity of the MgONPs displayed significant concentration-dependent inhibition via intimate contact with *R. solanacearum*. Furthermore, the MgONPs were adsorbed and dispersed on the bacterial cell walls, leading to destruction or disintegration of the cell walls, and then penetrated the bacterial cells, leading to leakage of the intracellular contents, which eventually resulted in cell death. The data showed that the destruction of the membrane integrity was related to physical injury and oxidation stress production on the bacteria or cell membrane. In addition, the production of DNA fragmentation and genotoxicity was probably connected with the reactive oxygen. The suppression of the swimming motility and twitching motility may indicate that the nanoparticles also decreased the biofilm formation of *R. solanacearum*, greatly reducing the bacterial infection of the host plants. In summary, we expect that MgONPs can present a promising alternative as antibacterial agents and can be extended to other phytopathogens in potential applications for controlling plant diseases in the future.

## Author Contributions

WD and LC designed the experiment. LC, JC, ZL, HW, and HY carried out the experiment. LC wrote the manuscript. WD and JC improved the manuscript. All authors read and approved the final manuscript.

## Conflict of Interest Statement

The authors declare that the research was conducted in the absence of any commercial or financial relationships that could be construed as a potential conflict of interest.
