# Supplementary material for: Magnesium Oxide Nanoparticles: Effective Agricultural Antibacterial Agent Against Ralstonia solanacearum
Source: Front Microbiol. 2018 Apr 25;9:790. doi: 10.3389/fmicb.2018.00790 (PMC5996892; doi:10.3389/fmicb.2018.00790)
Supplement: Supplementary file 1 [file Presentation_1.pdf]

*Supplementary Material*

# Magnesium oxide nanoparticles: Effective agricultural antibacterial agent against *Ralstonia solanacearum*

Lin Cai[1#], Juanni Chen[1#], Zhongwei Liu[2], Hancheng Wang[3], Huikuan Yang[1], Wei Ding[1*]

[*] Correspondence: Wei Ding Current address: College of Plant Protection, Southwest University, Beibei, Chongqing 400715, China. Email: **dwing818@163.com**

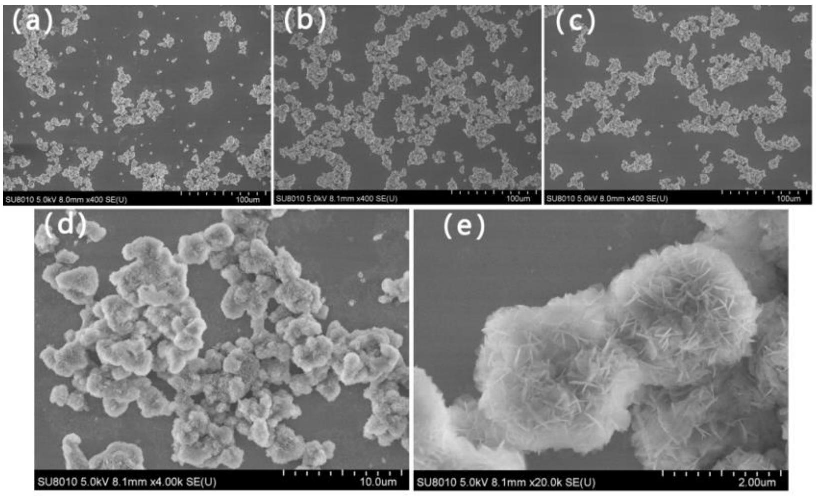


**Figure S1** SEM images of MgO NPs at different pH in the presence of water. (a) pH 5.0, (b) pH 7.0 and (c) pH 9.0; (d-e) part screen enlarged of (b).

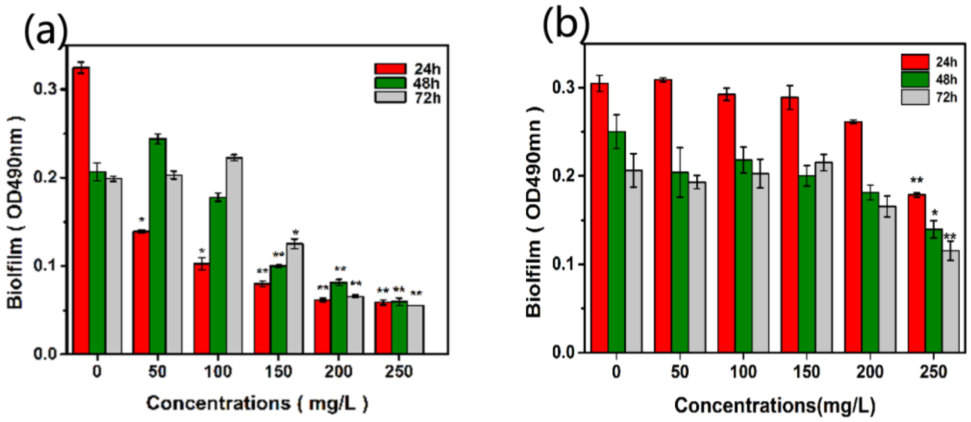


**Figure S2** Effect of MgONPs and bulk MgO on the biofilm formation of *R. solanacearum*. Treatments with the different concentrations of (a) MgONPs and (b) bulk MgO at 30 ℃ in the 96-well plates. The error bars in the histograms represent the standard deviation, and * and ** indicate $p < 0.05$ and $p < 0.01$, respectively.

**Table S1.** The minimum inhibitory concentrations and minimum bactericidal concentrations of the MgONPs, bulk MgO and thiodiazole copper against *R. solanacearum* in 96-well polystyrene microtiter plates.

| Antibacterial agent | MIC (mg/L) | MBC (mg/L) |
|---|---|---|
| MgONPs | 200 | 250 |
| Bulk MgO | 500 | 600 |
| thiodiazole copper | 125 | 200 |

.
